# Mr. Syme on Chloroform

**Published:** 1855-09

**Authors:** 


*Mr. Syme on Chloroform.*—So far as I can ascertain, from what I have heard and read upon the subject, there are important differences between the mode of administration of chloroform here and in London. It appears that here it is given according to principle, there according to rule. There, great attention is paid to the number of drachms or minims employed; here, we are entirly regardless of the amount used, and are guided only by the symptoms of the patient. The points that we consider of the greatest importance in the administration of chloroform, are—*First*, a free admixture of air with the vapor of the chloroform, to insure which, a soft porous material, such as a folded towel or handkerchief, is employed, presenting a pretty large surface, instead of a small piece of lint or any other apparatus held to the nose. *Secondly*, if this is attended to, the more rapidly the chloroform is given, the better, till the effect is produced; and hence we do not stint the quantity of chloroform. Then —and this a most important point—we are guided as to the effect, not by the circulation, but entirely by the respiration; you never see anybody here with his finger on the pulse while chloroform is given. So soon as the breathing becomes stertorous, we cease the administration; from what I have learned, it is sometimes pushed further elsewhere; but this we consider, in the highest degree, dangerous. *Thirdly*, *attention to the tongue* is another point which we find of great consequence. When respiration becomes difficult, or ceases, we open the mouth, seize the tip of the tongue with artery-forceps, and pull it well forward; and there can be little doubt that death would have occurred in some cases, if it had not been for this expedient. We also always give the chloroform in the horizontal position, and take care that there is no article of clothing constricting the neck. There are thus considerable differences between our practice and that which prevails, more or less, elsewhere. We use no apparatus whatever, take the respiration for our guide, attend to the condition of the tongue, and never continue beyond the point when the patient is fully under the influence of the anæsthetic.—*Lon. Lancet.*

